# Ultrafast Laser‐Induced Interatomic Forces in Magnetostrictive Metals

**DOI:** 10.1002/advs.202517754

**Published:** 2025-11-23

**Authors:** Xiaoxue Zeng, Lei Zhang, Yu Huang, Qingjie Fang, Yaokai Niu, Qiye Wen, Peng Yan, Zhifeng Chen, Zhiyong Zhong, Lichuan Jin

**Affiliations:** ^1^ State Key Laboratory of Electronic Thin Films and Integrated Devices University of Electronic Science and Technology of China Chengdu Sichuan 610054 China; ^2^ Research Center for Advanced Information Materials School of Physics and Materials Science Guangzhou University Guangzhou Guangdong 510006 China

**Keywords:** FeGa alloy, light‐induced interatomic forces, ultrafast strain birefringence

## Abstract

Femtosecond photoexcitation can abruptly redistribute electrons and trigger a series of transient nonequilibrium processes, among which ultrafast interatomic forces play a pivotal role in determining the structural and functional characteristics of solids. While ultrafast interatomic forces and their associated lattice dynamics have been extensively examined in semiconductors, experimental investigations of these nonequilibrium dynamics in metals remain lacking. To address this scientific gap, herein the direct observation of femtosecond‐scale variations in photoinduced ultrafast interatomic forces within wrinkled giant magnetostrictive FeGa thin films is presented. At the onset of demagnetization, a transient signal emerges, lasting ≈400 fs, with its orientation is influenced by the external magnetic field. Theoretical analysis indicates that this signal arises from the swift release of internal stress prompted by the suppression of magnetostriction during ultrafast demagnetization. Owing to magnetization‐induced stress anisotropy, this transient alteration in the interatomic potential introduces additional birefringence to the probe light. Consequently, this signal is attributed to a transient distortion of interatomic forces induced by the abrupt electron redistribution, establishing a nonequilibrium force state before any observable lattice expansion. These findings provide direct evidence for the existence of sub‐picosecond interatomic forces and suggest a novel approach to control metal lattice dynamics through ultrafast magnetostriction.

## Graphical abstract

This study reports ultrafast changes in the interatomic forces generated by femtosecond laser excitation in magnetostrictive FeGa thin films. This ultrafast process manifests as an optical birefringence signal lasting ≈400 fs. Using a pump‐probe system, the femtosecond evolution of this nonequilibrium interatomic interaction is visualized, providing new insights into ultrafast demagnetization and related atomic dynamics.

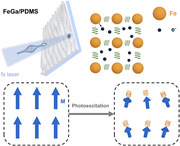

## 1 Introduction

Understanding the ultrafast response of materials following photoexcitation remains one of the most challenging and actively debated topics in contemporary physics.^[^ ^1^, ^2^, ^3^, ^4^, ^5^, ^6^, ^7^ ^]^ Femtosecond laser pulses are capable of dramatically reshaping the electronic structure of solids on ultrashort timescales, driving the system transiently far from equilibrium.^[^ ^8^, ^9^, ^10^, ^11^ ^]^ As electrons cross energy bands or redistribute within partially filled bands, shifts in electron occupancy alter the charge density distribution, leading to transient changes in various physical properties, including the dielectric constant,^[^ ^12^, ^13^, ^14^ ^]^ magnetization,^[^ ^15^, ^16^ ^]^ spin (orbital) angular momentum of the electron system,^[^ ^17^, ^18^, ^19^, ^20^ ^]^ and interatomic forces.^[^ ^21^, ^22^ ^]^ These electron‐related physical phenomena occur within the duration of excitation and within the subsequent tens to hundreds of femtoseconds—much faster than the few picoseconds required to convert electronic energy into thermal motion.^[^ ^22^, ^23^, ^24^ ^]^ This electron‐driven process challenges the traditional adiabatic view that atomic motion occurs along a fixed potential energy surface. The transient interatomic forces imbalance and the dynamic evolution resulting from ultrafast electronic excitation have profound implications for our understanding and control of the structural and functional responses of solids.

Semiconductors serve as ideal materials for investigating ultrafast changes in interatomic forces, primarily due to their well‐defined bandgaps and the significant influence of carrier excitation on bonding.^[^ ^25^, ^26^, ^27^ ^]^ Numerous investigations have demonstrated that exciting a substantial number of valence electrons into the conduction band is capable of transiently modifying the potential energy surface on sub‐picosecond timescales, thereby inducing marked changes in interatomic forces.^[^ ^28^, ^29^, ^30^ ^]^ Conversely, under the influence of interatomic forces, atoms promptly begin to move and rapidly acquire sufficient kinetic energy, resulting in coherent atomic motions and structural transformations, which enable ultrafast melting of the material on sub‐picosecond timescales.^[^ ^31^, ^32^, ^33^ ^]^ The energy of the electron is subsequently transferred to the lattice through electron‐phonon coupling, causing an increase in the phonon temperature and leading to thermal expansion or melting.^[^ ^34^, ^35^, ^36^ ^]^ Experimentally, studies of ultrafast interatomic forces typically utilize time‐resolved techniques such as time‐resolved transmittance or reflectance spectroscopy,^[^ ^21^ ^]^ X‐ray diffraction,^[^ ^37^, ^38^ ^]^ electron diffraction,^[^ ^39^, ^40^ ^]^ and two‐photon emission.^[^ ^41^ ^]^ These methodologies are able to effectively track the changes in various transient properties of a material following photoexcitation. However, similar investigations of ultrafast interatomic forces in metals present more challenges. Due to the absence of a bandgap, photoexcitation redistributes carriers within a continuum of electronic states, complicating the separation of interatomic forces from other concurrent effects. Furthermore, the strong electron‐phonon coupling in metals rapidly transfers electron energy to the lattice, obscuring the theoretically predicted sub‐picosecond interatomic forces imbalances. Therefore, most existing research remains largely theoretical^[^ ^42^, ^43^ ^]^ or it indirectly explores these phenomena using optical transmittance,^[^ ^44^, ^45^ ^]^ with direct experimental evidence being notably scarce.

In this work, we employ the time‐resolved pump–probe technique to optically investigate ultrafast magnetization and strain responses (see Notes S1 and S2, Supporting Information).^[^ ^46^ ^]^ A wrinkled FeGa film is triggered by a femtosecond near‐infrared laser pulse, after which the subsequent linearly polarized probe light traverses the sample, revealing a change in birefringence. This approach enables the simultaneous measurement of magnetization and the lattice states. The inherent stress gradient within the wrinkled FeGa film facilitates the separation of the interatomic forces signal from the magnetic signal response. Our experimental findings indicate that the interatomic forces are generated at the moment of demagnetization, manifesting as a distinct oscillation that is contingent on the external magnetic field. We quantitatively assess the time required for interatomic forces generation and decay, which is ≈400 fs, constrained by the resolution limits of our experiments. The interatomic forces induce variations in the dielectric tensor of the solid, effectively translating to an actual strain. The theoretical considerations demonstrate that this process elicits birefringence in the incident light, referred to as strain birefringence. Our research provides direct evidence for the presence of interatomic forces in metals, introduces a novel method for its detection, and paves the way for innovative lattice control in magnetostrictive systems.

## 2 Results and Discussion

**Figure 1:**
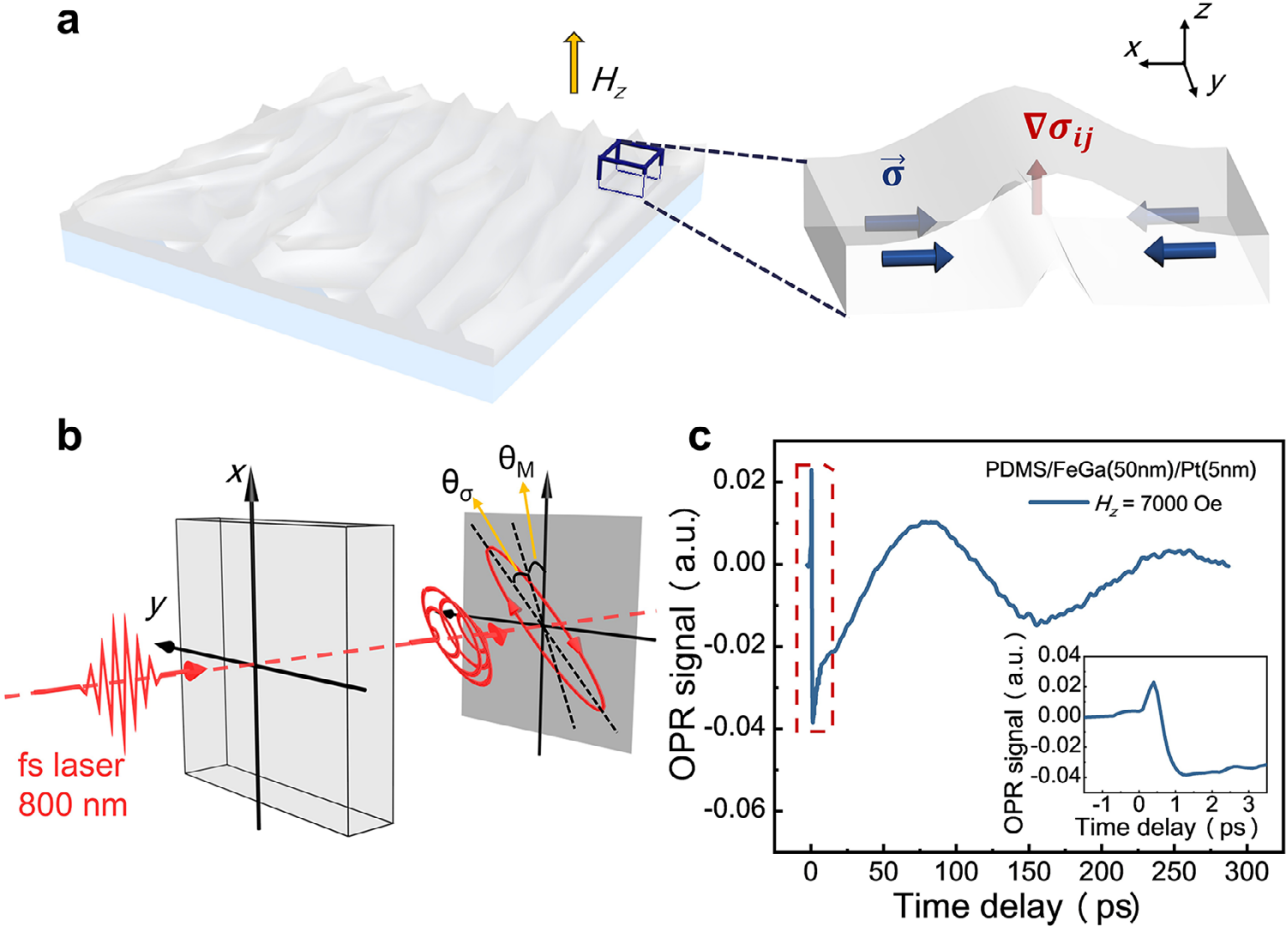
Schematic diagram of the sample and experimental setup. a) Wrinkled surface morphology of the PDMS/FeGa/Pt heterostructures, with stress (blue arrow) and stress gradient (red arrow) of the wrinkle. b) Schematic of light detection (see Figure S1, Supporting Information). Both magnetization (*M*) and stress (σ) will cause changes in the light polarization, with the detected light signal being the result of the superposition of two angles (θ_*M*_ + θ_σ_). c) OPR signal of PDMS/FeGa(50 nm)/Pt(5 nm) heterojunction subjected to an externally applied out‐of‐plane magnetic field (*H_z_* = 7000 Oe). Inset: details within the shown red dash box.

Here, we deposited FeGa (*t_FeGa_*)/Pt(5 nm) films on flexible polydimethylsiloxane (PDMS) substrates via magnetron sputtering, with *t_FeGa_*  = 10, 50, and 100 nm. The Pt layer is applied to the FeGa surface to protect it from oxidation. Below the Curie temperature (*T_C_* = 712.8 K),^[^ ^47^ ^]^ the FeGa alloy exhibits ferromagnetism along with a strong inverse magnetostrictive effect. During the thin‐film deposition process, the substrate temperature rises, resulting in a significant thermal expansion coefficient mismatch between the film and substrate. Consequently, upon cooling, the substrate contracts, exerting compressive stress on the film and leading to the development of wrinkles (see **Figure** **1**a).^[^ ^48^, ^49^, ^50^ ^]^ We characterized the surface morphology, magnetic properties, and crystal structure of the PDMS/FeGa/Pt structure with varying FeGa thicknesses (see Note S3, Supporting Information). The film is predominantly amorphous, exhibiting a multitude of random surface wrinkles. Notably, the magnetic moment at the apex of these wrinkles typically presents a magnetization component oriented normal to the film plane (along the +**z** axis in Figure 1a). Additionally, there is a positive correlation between the magnitude of the perpendicular magnetization component and the height of wrinkles (see Figure S3, Supporting Information).

We begin by investigating the ultrafast stress response in the PDMS/FeGa (50 nm)/Pt (5 nm) structure utilizing the femtosecond time‐resolved pump–probe method. The observed variation in the polarization angle of the detected light is primarily attributed to contributions from both magnetism and lattice dynamics (see Figure 1b). The optical polarization rotation (OPR) signal exhibits a characteristic ultrafast demagnetization process that encompasses demagnetization and relaxation, accompanied by magnetization precession (see Figure 1c). In FeGa films, the magnetization precession arises from the transient reorientation of the effective magnetic field caused by laser‐induced modifications of the magnetic anisotropy through thermal and magnetoelastic effects (see Note S4, Supporting Information). Notably, at the onset of demagnetization, an anomalous transient signal appears in the signal trace (see inset of Figure 1c).

**Figure 2:**
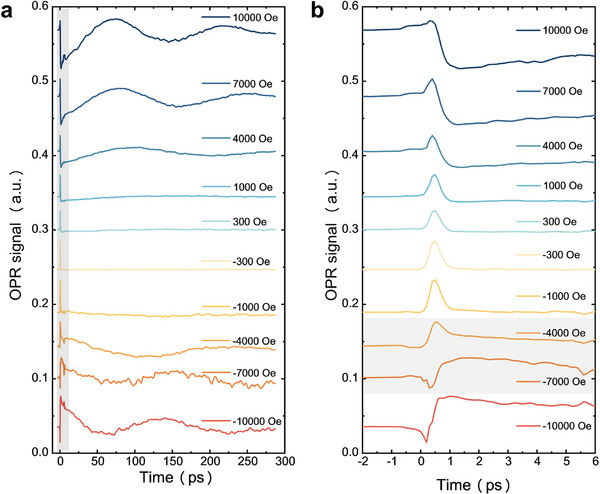
OPR raw signals of the PDMS/FeGa(50 nm)/Pt(5 nm) structure. a) The OPR signals subjected to varying externally applied out‐of‐plane magnetic field, with the laser incident from the Pt surface. b) Enlarged view of the gray area in panel (a), where within the light‐gray area, the stress signal exhibits a reversed sign.

We examined the probe light signal by varying the externally applied out‐of‐plane magnetic field, *H_z_* (see **Figure** **2**a). The experimental results reveal that the transient signal persists under varying strengths of the applied magnetic field. To elucidate the origin of this ultrafast signal, as shown in Figure 2b, we analyzed the demagnetization process with high temporal resolution. Remarkably, the ultrafast transient signal is observed to reverse within the range of *H_z_* = −4000 to −7000 Oe. We further reversed the sample and conducted the same measurements (see Note S5, Supporting Information). When the laser is incident from the PDMS substrate side, the sign of the ultrafast transient signal inverts within the range of *H_z_* = +1000 to +4000 Oe. This observation substantiates the authenticity of the ultrafast transient signal and indicates a correlation with the internal bias magnetic field in the normal direction (along the +**z** axis in Figure 1a) of the film.

Moreover, we observed that the internal bias magnetic field associated with the signal reversal is enhanced when the light is incident from the sample surface, in contrast to when it is incident from the substrate side. This phenomenon will be elaborated upon in detail below. The time‐domain analysis reveals that the transient signal appears ≈400 fs after the initiation of demagnetization and subsequently decays within the following 400 fs. This rapid change cannot be ascribed to magnetization precession,^[^ ^51^, ^52^, ^53^ ^]^ which is typically observed following demagnetization in ferromagnetic materials, nor to lattice dynamics^[^ ^35^, ^36^ ^]^ resulting from magnetoacoustic coupling. Furthermore, the observed regularity and the dependence of the transient signal on the externally applied magnetic field rule out the possibility of coherent artifact signals that could be significantly influenced by the optical measurement setup.

**Figure 3:**
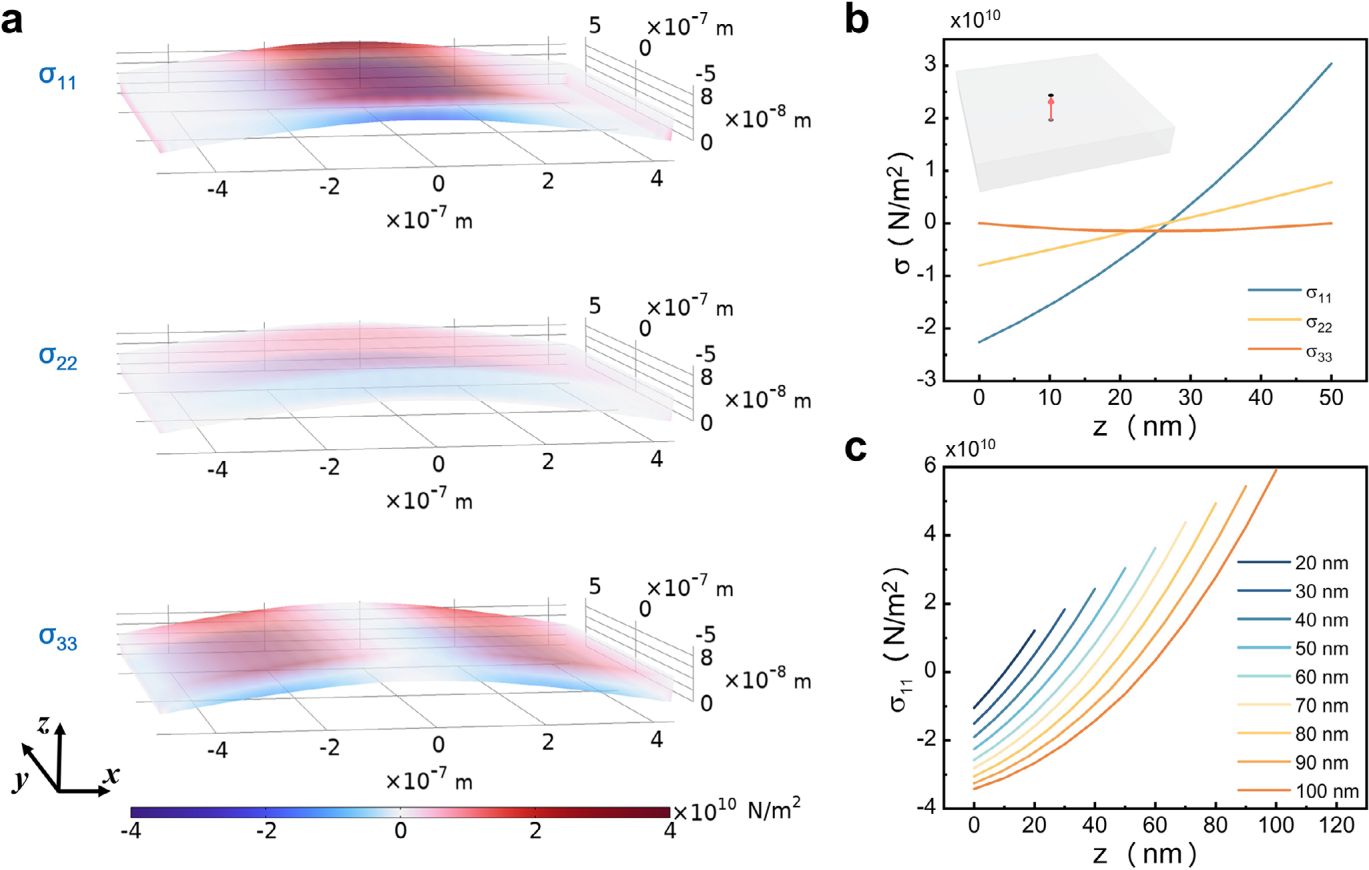
COMSOL simulations of the normal stresses in various directions within a bent film. a) Distribution of stress components in a 50 nm thick bent film, with red indicating tensile stress and blue indicating compressive stress. b) Variation of the normal stress component at the center of a 50 nm thin film as a function of the depth within the film. The red line in the inset denotes the baseline used for data extraction. c) The x‐component of tensile stress along the baseline in films of varying thicknesses.

Our objective is to elucidate the physical mechanism underlying the transient signal, which is influenced by the externally applied magnetic field. Therefore, it is essential to investigate the origin of the internal bias magnetic field. The results differ from those observed on a hard substrate, indicating that this phenomenon may originate from the wrinkled morphology of the film.^[^ ^54^ ^]^ To further explore the origin of the internal bias magnetic field in the wrinkled film, we calculated the internal stress distribution using COMSOL Multiphysics simulations (see Note S6, Supporting Information). The film dimensions were set to 1 µm × 1 µm × *t_FeGa_* (*t_FeGa_*  =  20−100 nm). All material parameters are derived from the actual characteristics of the FeGa film. Considering the random and complex nature of the film's wrinkle morphology, we initially examined the idealized scenario of uniform bending. The model presupposes uniform film growth, succeeded by the contraction of one edge toward the film's center after growth completion. The simulation results elucidate the internal stress distribution within the bent film. We illustrated the normal stress components σ_*ii*_ in various directions, as depicted in **Figure** **3**a. Our primary focus was on the center of the bent film, where we experimentally measured the internal bias magnetic field. In this central region, the stress manifests predominantly as normal stress along the x‐axis and y‐axis. The upper surface experiences tensile stress, whereas the lower surface endures compressive stress, culminating in a stress gradient along the *z*‐axis. Importantly, there exists a notable discrepancy between stress magnitudes associated with the x‐axis and y‐axis, signifying in‐plane anisotropy.

We analyzed the stress distribution along the normal direction at the center of a 50 nm film as indicated by the red arrow in the inset of Figure 3b, the main obtained results are illustrated in Figure 3b. When the film is subjected to bending stress, the internal stress in the central region predominantly arises along the *x*‐ and *y*‐directions. For films of various thicknesses, σ_11_ exhibits a significant stress gradient in the normal direction (see Figure 3c). We employed the same methodology to determine the shear stress component (see Note S6, Supporting Information). Under the same conditions, the shear stress at the center of the film is approximately ten orders of magnitude lower than the normal stress, with the shear stress in the *xz‐*direction σ_13_ demonstrating an antisymmetric nature.

The COMSOL simulation results suggest that the internal bias magnetic field primarily stems from the normal stress gradient. This gradient disrupts the symmetry of the film in the normal direction, consequently inducing an internal bias magnetic field (see Note S2.1, Supporting Information). Given that the COMSOL model is unable to simulate large‐angle bending of the film, we could not obtain quantitative data from simulations. Nonetheless, the qualitative correlation between the simulated stress gradient and the observed phenomena provides substantial support for our proposed mechanism. To quantify this, we have developed a theoretical model to articulate the internal bias magnetic field:

(1) $$H_{\sigma ,\;z}=\frac{3\lambda_{s}}{2\mu_{0}M_{s}}\;\frac{\partial (\sigma_{11}\left(z\right)+\sigma_{22}\left(z\right))}{\partial z}\Delta z,$$

where λ_*s*_ represents the magnetostriction coefficient, and *M_s_* denotes the saturation magnetization. This induces spontaneous magnetization at the top of the bent film and leads to additional film deformation due to the magnetostrictive effect.

When a femtosecond laser pulse penetrates a ferromagnetic metal film, it excites d electrons, leading to the formation of a nonequilibrium electron gas,^[^ ^3^, ^15^ ^]^ which subsequently induces magnetization and lattice dynamics. This energy dissipation process is typically described by the phenomenological three‐temperature model.^[^ ^15^ ^]^ This model conceptualizes the system as consisting of three interdependent subsystems: electrons, lattice, and spin, and it characterizes the energy variations of these subsystems through their time‐dependent temperatures. Alterations in the electronic occupation distribution give rise to ultrafast interatomic forces and demagnetization,^[^ ^55^, ^56^ ^]^ which align with the time scales of the electron temperature and magnetization as outlined in the three‐temperature model, respectively. The general three‐temperature model can be expressed as:

(2) $$\begin{array}{c} C_{e}\left(T_{e}\right)\;\frac{dT_{e}}{dt}=\nabla_{z}\;\left(\kappa_{e}\left(T_{e}\right)\nabla_{z}T_{e}\right)+g_{ep}\left(T_{p}-T_{e}\right)+S\left(z,t\right),\\ C_{p}\left(T_{p}\right)\;\frac{dT_{p}}{dt}=\nabla_{z}\left(\kappa_{p}\left(T_{p}\right)\nabla_{z}T_{p}\right)+g_{ep}\;\left(T_{e}-T_{p}\right),\\ \;\frac{dm}{dt}=Rm\frac{T_{p}}{T_{C}}\left(1-m\;coth\left(\frac{mT_{C}}{T_{e}}\right)\right), \end{array}$$

where (*C_e_*, *T_e_*) and (*C_p_*, *T_p_*) are the heat capacities and temperature of electron and lattice reservoirs, respectively. In each subsystem, the heat capacity is proportional to the system temperature. ∇_*z*_ denotes the differentiation with respect to *z*, and κ represents the thermal conductivity. In addition, *g_ep_* stands for the coupling constant that characterizes the rate of energy exchange between the subsystems. *T_C_* denotes the Curie temperature, and R is associated with the demagnetization ratio of the material. Further, *m*  =  *M*/*M_s_* represents the magnetization relative to its saturation value. *S*(*z*, *t*) is the laser source term describing the instantaneous electronic disturbance by a femtosecond laser pulse.

**Figure 4:**
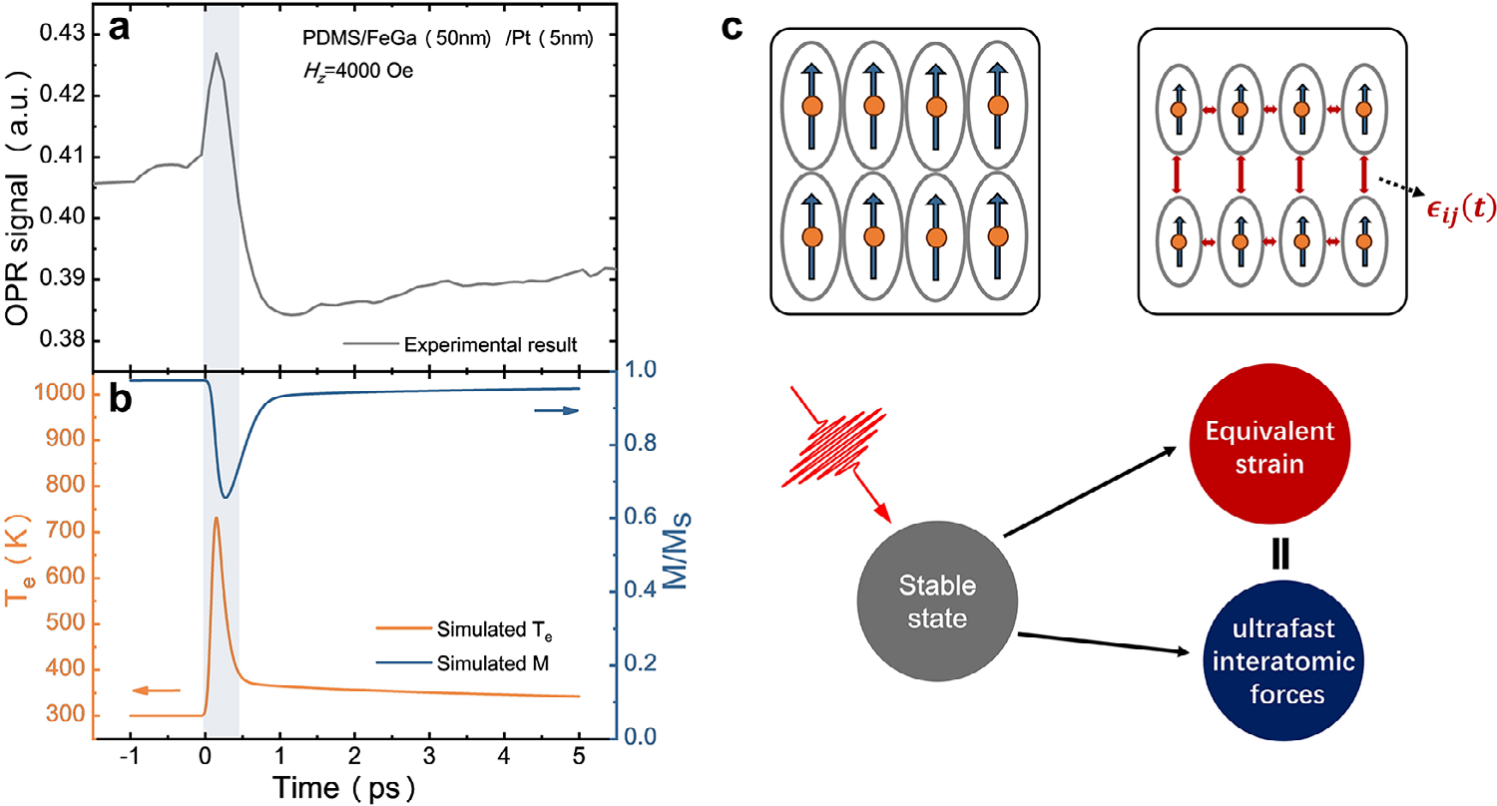
Ultrafast magnetoelasticity dynamics of the transient signal. a) OPR signal of the PDMS/FeGa(50 nm)/Pt(5 nm) heterojunction under the externally applied out‐of‐plane magnetic field (*H_z_* = 4000 Oe). b) Electron temperature (*T_e_*, orange) and magnetization (*M*, blue) as a function of time delay in the PDMS/FeGa(50 nm)/Pt(5 nm) sample. c) Schematic diagram of the interatomic forces generated process.

We modeled the ultrafast transient signal in a 50 nm FeGa film using a simplified 1D model, leveraging the Python toolbox *udkm1Dsim*.^[^ ^57^ ^]^ This methodology provided deeper insights into the dynamics of the process by enabling the calculation of the electron temperature (*T_e_*), lattice temperature (*T_p_*), and magnetization *M* (see details in Note S2.2, Supporting Information). The model was utilized to compute the temporal variations of *T_e_* (orange) and *M* (blue), as illustrated in **Figure** **4**b. The obtained results are indicative of the fact that the sudden changes in *T_e_* are primarily related to specific intervals when *M* experiences a significant decrease. This observation aligns with prior theoretical findings suggesting that demagnetization is caused by d electron transitions. A comparison between the experimental data presented in Figure 4a, illustrates that the magnetic field‐dependent ultrafast signal correlates with alterations in *T_e_*, thereby confirming that this ultrafast signal indeed corresponds to changes in electronic behavior. It is noteworthy that the simulation's magnetization deviates from the experimental observations results. This discrepancy can be attributed to the increase in the lattice temperature during the relaxation process, which induces a random orientation of the magnetized units.^[^ ^58^ ^]^ The magnetic moments necessitate the continuous precession to realign with the sign of the effective magnetic field, as described by the Landau—Lifshitz–Bloch (LLB) equation.^[^ ^51^, ^52^ ^]^ Additionally, the three‐temperature model^[^ ^57^ ^]^ does not account for the nonequilibrium electron–electron scattering processes,^[^ ^13^, ^14^ ^]^ instead employing the jump function as the initial condition, which may contribute to simulation inaccuracies.

The conclusions drawn above indicate that the dependence of the transient signal on the externally applied magnetic field arises from the stress distribution within the film, thereby confirming that this ultrafast transient signal is indeed a strain signal. As detailed in Note S2.4, Supporting Information, variations in the electronic occupation distribution give rise to a nonequilibrium state in the interatomic forces. This nonequilibrium state is manifested itself as anisotropic changes in the dielectric tensor, resulting in the transient birefringence of the transmitted light. This can be mathematically stated by

(3) $$\begin{array}{c} \Delta \varepsilon_{ij}\;\left(t\right)=a_{1}\;u_{ll}\left(t\right)\delta_{ij}+a_{2}u_{ij}\left(t\right)\\ \theta_{prob-e}\propto \Delta \varepsilon_{ij}\left(t\right) \end{array}$$

where the coefficients *a*_1_ and *a*_2_ represent elastic‐optical constants. In addition, *u_ll_* is isotropic volumetric strain due to volume expansion, and *u_ij_* is anisotropic contribution from the strain tensor, including both normal and shear components. Δε_*ij*_(*t*) is the time‐dependent change in the dielectric tensor. Finally, θ_*prob* − *e*_ donates the optical polarization angle induced by the photoelastic effect. This ultrafast signal primarily emanates from the regions adjacent to the center of the film wrinkles. A schematic illustration of the laser‐induced ultrafast interatomic forces is presented in Figure 4c. Upon light excitation, *M* experiences an instantaneous reduction. Concurrently, the magnetostriction coefficient undergoes a corresponding alteration, which can be phenomenologically represented by^[^ ^59^, ^60^ ^]^

(4) $$\lambda \propto \lambda_{s}{\left(\frac{M}{M_{S}}\right)}^{2}$$

The drastic reduction in the magnetostriction coefficient during demagnetization is the main reason for the generation of ultrafast interatomic forces in giant magnetostrictive FeGa films. In the following passage, we will describe the dynamics of this process within a wrinkle in some detail.

**Figure 5:**
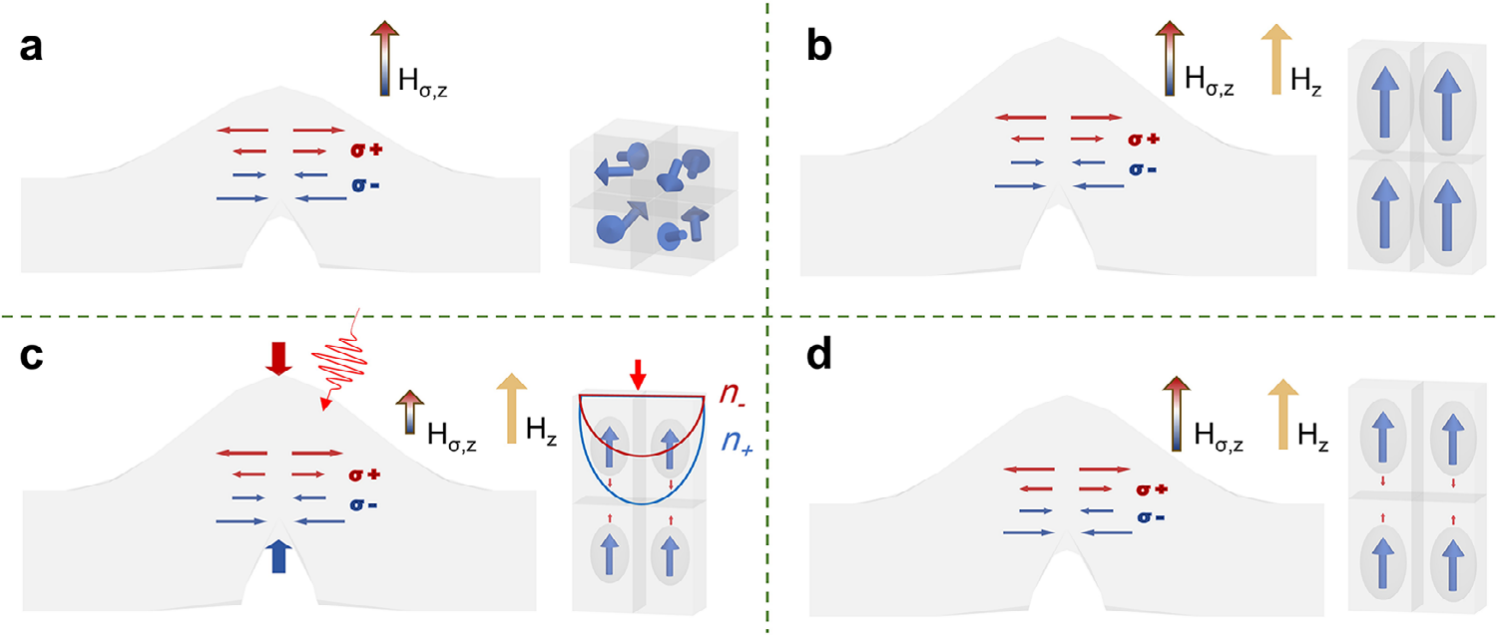
Ultrafast interatomic forces induced by femtosecond laser. a) Distribution of stress and magnetic moment orientation in the bent film initial state. b) The film increases in the normal dimension due to an externally applied out‐of‐plane magnetic field. c) Ultrafast interatomic forces induced by laser excitation, which results in optical birefringence (*n*_±_). d) Relaxation and disappearance of the interatomic forces (*note*: On the left side of each subfigure is a schematic diagram of the bent film, where the red and blue arrows represent normal tensile stress (σ^+^) and compressive stress (σ^−^), respectively. On the right side, there is a microscopic diagram).

a) **Initial State**. The magnetic moment distribution within the film is predominantly governed by the shape anisotropic field and the effective magnetoelastic field. At the crest of the wrinkle, the internal stress gradient generates an internal bias magnetic field (*H*_σ,*z*_), in the normal direction, as illustrated in **Figure** **5**a. In the majority of regions outside the wrinkles, the magnetic moments are oriented randomly, forming distinct magnetic domains.

b) **Magnetization State**. In the central region of the wrinkled film, the magnetic moment exhibits out‐of‐plane anisotropy due to the influence of the internal bias magnetic field. The interplay between the internal bias magnetic field and the externally applied magnetic field in the middle of the wrinkle further modifies the interatomic forces and the overall magnetization state (see Figure 5b).

c) **Ultrafast Demagnetization and Magnetic Elasticity**. Femtosecond laser excitation elevates orbital electrons to a high‐energy state, resulting in the generation of hot electrons. Alterations in the electronic occupation distribution leads to demagnetization and disrupt the balance of the interatomic forces,^[^ ^55^, ^61^, ^62^ ^]^ as depicted in Figure 5c. In the course of the ultrafast demagnetization process, the contribution of magnetostriction in the direction of magnetization direction is significantly reduced. The prominence of internal stress within the film to becomes evident. The effects of this transient nonequilibrium resemble actual atomic displacement, directly resulting in change in the dielectric tensor within the solid. The anisotropy of magnetostriction could induce anisotropy in the transient interatomic forces, thus making the dielectric tensor also anisotropic, which leads to a linear birefringence for light propagating along the optical *z*‐axis (see Note S2.4, Supporting Information).^[^ ^46^, ^63^ ^]^ In planar thin‐film materials, ultrafast stress typically exhibits bidirectional anisotropy without symmetry breaking. However, for films with a wrinkled morphology, the internal stress gradient introduces a symmetry breaking effect along the normal direction, resulting in outcomes that depend on the external magnetic field.

d) **Relaxation process**. Excited electrons release energy by transferring it to phonons and spins, leading to a realignment of magnetization that aligns with the direction of the effective magnetic field. This process is often accompanied by dynamic phenomena, such as magnetic moment precession^[^ ^64^ ^]^ and the excitation of phonon groups^[^ ^36^ ^]^ (see Figure 5d). We detected ultrafast interatomic forces signals in a sample with a 10 nm‐thick layer using the same methodology (see Note S7, Supporting Information). In samples with thinner FeGa layers, the interatomic forces signals appear relatively diminished. Under strong external magnetic fields, distinguishing these signals from the demagnetization signals becomes increasingly challenging. Conversely, under weaker external magnetic fields, distinct transient signals remain observable. This observation can be attributed to the reduced internal stress that accumulates during the deposition process in thinner films, resulting in less prominent effects from light‐induced interatomic forces.

We conclude by systematically addressing and eliminating alternative sources of the observed ultrafast transient signal:

First, the transient signal detected in the wrinkled sample exhibits stability, with a discernible oscillation state consistently identified across samples of varying thicknesses. Furthermore, the transient signal's polarity is influenced by the external magnetic field. We recorded the PDMS substrate signal, which is more than an order of magnitude weaker than that of the wrinkled sample and remains unchanged in the presence of the external magnetic field (see Note S8, Supporting Information). Utilizing the same methodology, we investigated the signal from the MgO/FeGa (10 nm)/Pt (5 nm) sample and similarly did not observe any signal reversal (see Note S9, Supporting Information). This allows us to reasonably discount the possibility of coherent optical artifacts as a contributing factor.

Second, we meticulously eliminated electronic artifacts as a source of interference. The electron distribution state immediately following the pump laser's interaction with the sample surface substantially influences optical transmittance and reflectance changes, commonly referred to as “electronic artifacts”. Prior investigations have almost attributed such ultrafast alterations to the influence of interatomic forces on the dielectric constant.^[^ ^44^, ^45^ ^]^ We measured the transmittance of PDMS/FeGa(50 nm)/Pt (see Note S10, Supporting Information), and the results demonstrated that the sign of the transmittance signal does not reverse with variations in the external magnetic field. This suggests that the ultrafast signal we observed, which depends on the external magnetic field, does not stem from direct changes in optical transmittance; rather, it arises from a birefringence effect instigated by interatomic forces.

Third, we excluded the effects of the strain wave induced by lattice thermal expansion. According to the three‐temperature model, lattice thermal expansion is mostly attributed to electron‐phonon coupling, where electron energy dissipation leads to the generation of phonon modes.^[^ ^56^ ^]^ Lattice temperature changes typically manifest as lattice dynamics lasting a few to a few hundred picoseconds.^[^ ^36^ ^]^ This suggests that the observed signal is a transient, non‐thermal process, rather than a thermal process.

Lastly, prior investigations have shown that magnetization precession occurs solely during the relaxation process, usually beyond 20 ps.^[^ ^53^ ^]^ The magnetization precession frequency in FeGa alloy films is ≈34 GHz, significantly lower than the rapid variation rate of the transient signal (see Figure 2a). Consequently, we can also eliminate signals attributed to ultrafast magnetization dynamics as a source of interference.

## 3 Conclusion

In summary, we present experimental evidence for photoinduced interatomic forces in metallic FeGa thin films. These ultrafast interatomic forces result from transient alterations in the electron distribution induced by photoexcitation. Due to the material's significant magnetostriction coefficient, the dielectric constant variation induced by the interatomic forces in the FeGa film exhibits anisotropy along the magnetization direction, manifesting as a birefringence signal that persists for ≈400 fs. The experiments demonstrate that the ultrafast interatomic forces in the FeGa film correlates with the stress distribution within the film, suggesting that the lattice interatomic forces are equivalent to actual strain. Our results provide compelling evidence for the existence of nonthermal interatomic forces in metals and introduce a novel method for assessing this ultrafast nonequilibrium forces. From an application standpoint, interatomic forces hold considerable promise, particularly for the investigation and manipulation of ultrafast lattice dynamics, thereby opening new avenues for the development of ultrafast magnetic and acoustic devices.

## 4 Experimental Section

### Sample Fabrication

The FeGa/Pt samples were deposited on PDMS and MgO substrates via FeGa alloy and Pt targets for magnetron sputtering at room temperature. Unless stated otherwise, the base pressure was maintained at 1 × 10^−5^ Pa, and the Ar pressure was set as 0.3 Pa. The substrate dimensions used were 10 mm × 10 mm × 0.5 mm.

### Time‐Resolved Magnetic‐Optical Faraday Measurement

The ultrafast laser‐induced OPR measurement was performed in a home‐built pump‐probe spectroscopy system. Femtosecond laser pulses generated by a Ti:sapphire regenerative amplifier, characterized by a central wavelength of 800 nm, a pulse duration of 100 fs, a repetition rate of 1 kHz, and a fluence of 2.5 mJ cm^−2^, were used to pump the magnetic multilayers.^[^ ^65^, ^66^, ^67^, ^68^ ^]^ A weak linearly polarized light beam traversed the film, was split by a Wollaston prism, and then entered a balanced detector. The differential signal obtained from the balanced detector is directly proportional to the polarization rotation (birefringence) imparted to in the probe beam by the sample.

### Pump‐Probe Transmission spectrum

The transmission spectrum was acquired utilizing a home‐built pump‐probe spectroscopy system. The laser parameters were consistent with those described previously. Both the pump and probe beams traversed through the delay line and were focused onto the same point on the sample, achieving a spot size of ≈100 µm. After passing through the sample, the probe beam directly entered the photodetector directly. The output signal from the detector represented the instantaneous intensity of the transmitted light.

### Statistical Analysis

The experimental samples were measured repeatedly utilizing a pump‐probe system. Each curve displayed represents the average of these repeated measurements, computed with Origin software. A sample size of 10 was used for the statistical analysis of each curve.

## Conflict of Interest

The authors declare no conflict of interest.

## Supporting information



Supporting Information

## Data Availability

The data that support the findings of this study are available from the corresponding author upon reasonable request.
