# Supplementary material for: Ultrafast Laser‐Induced Interatomic Forces in Magnetostrictive Metals
Source: Adv Sci (Weinh). 2025 Nov 23;13(8):e17754. doi: 10.1002/advs.202517754 (PMC12884780; doi:10.1002/advs.202517754)
Supplement: Supplementary file 1 — Supporting Information [file ADVS-13-e17754-s001.docx]

**Supplementary Information**

This Supplementary Information file contains:

Supplementary Note 1: Experimental Setup

Supplementary Note 2: Modelling of the ultrafast magnetoelastic effect

Supplementary Note 3: Material characterization

Supplementary Note 4: Magnetization precession during relaxation

Supplementary Note 5: The OPR signals when laser incident from PDMS substrate

Supplementary Note 6: COMSOL simulation: model setup and shear stress results

Supplementary Note 7: Substrate incident test results of $t_{FeGa}$ = 10 nm

Supplementary Note 8: Test results of PDMS substrate

Supplementary Note 9: Test results of MgO/FeGa(10 nm)/Pt

Supplementary Note 10: Transmission rate of PDMS/FeGa(50nm)/Pt

**Supplementary Note 1: Experimental Setup**


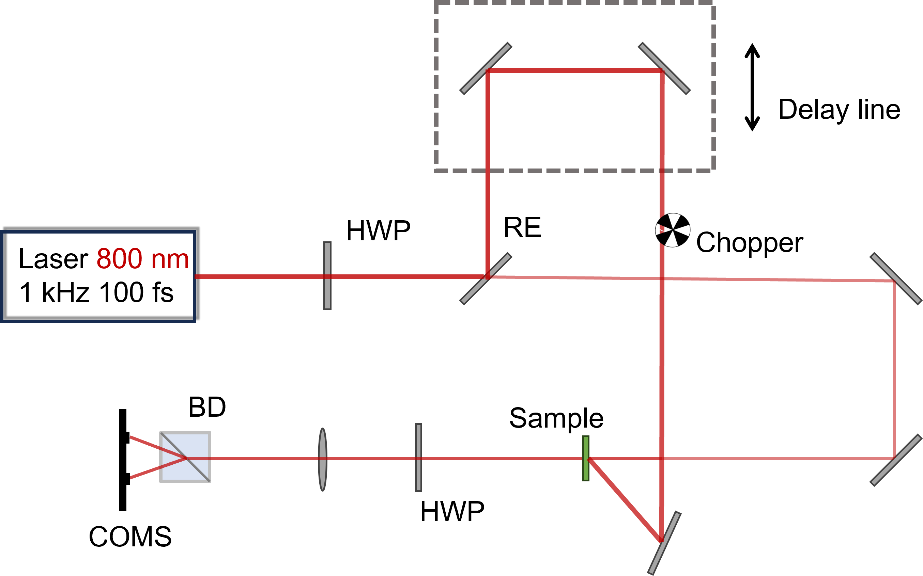


**Fig. S1 Schematic diagram of time-domain pump—probe technique.** HWP: half wave plate, RE: 95% reflector, BD: calcite beam displacer.

Figure S1 shows a simplified schematic of the experimental optical path. Linearly polarized femtosecond laser pulses were generated by the femtosecond laser system. The pump femtosecond pulses passed through a delay line and a chopper before being incident on the sample. The probe laser pulses then passed through the sample, with a spot size of 100 μm. The OPR signal was detected using optical balanced bridge.

**Supplementary Note 2: Modelling of the ultrafast magnetoelastic effect**

In this section, we present the theoretical models related to the generation and detection of ultrafast magnetoelasticity effects.

The FeGa alloy film grown on PDMS flexible substrates exhibits a random wrinkled morphology, making it challenging to describe these details. For simplicity, we consider a regularly bent film, consistent with the COMSOL simulation.

**S2.1. Stresses in FeGa film**

FeGa alloy has strong magnetostriction properties.^[1, 2]^ In the wrinkled FeGa film, both the internal stress induced by the shrinkage of the PDMS substrate and the magnetostrictive effect must be considered. Based on the experimental and simulation results, we focus on the regions with perpendicular magnetization component at the middle of the wrinkled film. At this point, the normal stress exhibits a gradient distribution across the film thickness, resulting in the following stress matrix form:

|  | $\boldsymbol{\sigma(z)}=\left( \begin{matrix} \sigma_{11}(z) & 0 & 0 \\ 0 & \sigma_{22}(z) & 0 \\ 0 & 0 & \sigma_{33}(z) \end{matrix} \right).$ | (S1) |
| --- | --- | --- |

For numerical and symmetry reasons, shear stresses can be neglected, and only normal stresses in the in-plane direction are taken into account. The total energy density of the film can be described as:

|  | $E_{total}=E_{mag}+E_{\sigma}+E_{el},$ | (S2) |
| --- | --- | --- |

where $E_{mag}$ represents the energy density term, which includes contributions from exchange interaction, demagnetization, anisotropy and external magnetic field. $E_{\sigma}$ denotes the mechanical energy, while $E_{el}$ represents the magnetoelastic energy, including the magnetoelastic contribution. In the absence of an external magnetic field, the orientation of the magnetic moments in the magnetostrictive material is influenced by the stress distribution within the film. Considering the in-plane gradient distribution in the z-axis direction, the magnetoelastic energy can be expressed as:

|  | $E_{\sigma}\left( z \right)=-\frac{3}{2}\lambda_{s}\left( \sigma_{11}\left( z \right)\cdot\cos^{2} \theta_{1}+\sigma_{22}\left( z \right)\cdot\cos^{2} \theta_{2} \right),$ | (S3) |
| --- | --- | --- |

where $\lambda_{s}$ is magnetostriction coefficient. In polycrystalline materials, the magnetostriction coefficient is given by $\lambda_{s}=\lambda_{100}=\lambda_{111}$.^[3]^ $\theta_{1}$ and $\theta_{2}$ are the angles between the magnetization $M$ and the stresses $\sigma_{11}$ and $\sigma_{22}$. After considering the gradient of the normal stress in the x- and y-axes direction, the magnetoelastic energy is expressed as:

|  | $\frac{\partial E_{\sigma}(z)}{\partial z}=-\frac{3}{2}\lambda_{s}\frac{{\partial(\sigma}_{11}\left( z \right)\cdot\cos^{2} \theta_{1}+\sigma_{22}\left( z \right)\cdot\cos^{2} \theta_{2})}{\partial z}.$ | (S4) |
| --- | --- | --- |

Therefore, considering the magnetoelastic coupling, the equivalent magnetic field is given by:

|  | $\begin{matrix} \boldsymbol{H}_{\sigma, eff}=-\frac{1}{\mu_{0}M_{S}}\frac{\partial E_{\sigma}\left( z \right)}{\partial M} \\ =\frac{3\lambda_{s}{(\sigma}_{11}(z)+\sigma_{22}\left( z \right))}{2\mu_{0}M_{s}}\boldsymbol{u}_{x}+\frac{3\lambda_{s}}{2\mu_{0}M_{s}}\frac{\partial{(\sigma}_{11}(z)+\sigma_{22}\left( z \right))}{\partial z}\Delta z\boldsymbol{u}_{z}. \end{matrix}$ | (S5) |
| --- | --- | --- |

That is, stress gradient can induce an equivalent bias perpendicular magnetic field at the center of the wrinkle, which aligns the magnetic moment normal to the film plane. Although the deformation caused by magnetostriction is negligible compared to the film contraction deformation, it must be considered after the wrinkle center is magnetized, as it leads to a localized increase in film thickness. Assuming the region is fully perpendicularly magnetized, the resulting magnetostrictive strain can be estimated as follows:

|  | $\Delta t_{FeGa}=t_{FeGa}\cdot\lambda_{s}.$ | (S6) |
| --- | --- | --- |

When the material reaches a stable state, the film strain can be expressed as:

|  | $\boldsymbol{\epsilon}=\left( \begin{matrix} \epsilon_{11}\left( z \right)+\Delta\epsilon_{11} & \Delta\epsilon_{12} & \Delta\epsilon_{13} \\ \Delta\epsilon_{21} & \epsilon_{22}(z)+\Delta\epsilon_{22} & \Delta\epsilon_{23} \\ \Delta\epsilon_{31} & \Delta\epsilon_{32} & \epsilon_{33}\left( z \right)+\Delta\epsilon_{33} \end{matrix} \right),$ | (S7) |
| --- | --- | --- |

where $\epsilon_{ij}\left( z \right)=\sigma_{ij}(z)/E$, and $E$ is the Young's modulus. $\Delta\epsilon_{ij}$ is the additional strain caused by magnetostriction in the film, and $\Delta\epsilon_{33}=\Delta t_{FeGa}$.

**S2.2. Ultrafast magnetoelastic effect during electronic state change**

When a femtosecond laser pulse strikes the material, the outer orbital electrons of the metal atoms will be excited and form a hot electron distribution, which disrupt the interaction between atoms and induce ultrafast stress. As the magnetic moment reduces during the demagnetization process, the effect of the equivalent bias magnetic field induced by the stress gradient diminishes. Concurrently, the significant normal stress in the in-plane direction becomes more pronounced, triggering ultrafast lattice dynamics. This process can be described by a three-temperature model.^[4]^ The ultrafast demagnetization process reflects the change in the effective magnetic moment. Ultrafast stress, on the other hand, is related to electron temperature because it directly corresponds to the electron distribution state. The general three-temperature model can be expressed as:

|  | $C_{e}\left( T_{e} \right)\frac{dT_{e}}{dt}=\nabla_{z}\left( \kappa_{e}\left( T_{e} \right)\nabla_{z}T_{e} \right)+g_{ep}\left( T_{p}-T_{e} \right)+S\left( z,t \right),$ | (S8) |
| --- | --- | --- |
|  | $C_{p}\left( T_{p} \right)\frac{dT_{p}}{dt}={\nabla_{z}\left( \kappa_{p}\left( T_{p} \right)\nabla_{z}T_{p} \right)+g}_{ep}\left( T_{e}-T_{p} \right),$ | (S9) |
|  | $\frac{dm}{dt}=Rm\frac{T_{p}}{T_{C}}\left( 1-m coth\left( \frac{mT_{C}}{T_{e}} \right) \right),$ | (S10) |

where $(C_{e}, T_{e})$ and ${(C}_{p}, T_{p})$ are heat capacities and temperature of electron and lattice reservoirs. $\nabla_{z}$ denotes differentiation with respect to *z*, and $\kappa$ is the thermal conductivity. The electron heat capacity is given by $C_{e}=\gamma T_{e}$. $g_{ep}$ is coupling constant that describe the rate of energy exchange between the subsystems. $S(z,t)$ is the laser source term describing the instantaneous electronic disturbance by a femtosecond laser pulse. $T_{C}$ is the Curie temperature, and R is related to the demagnetization ratio of the material. $m=M/M_{s}$ is the magnetization relative to its saturation value.


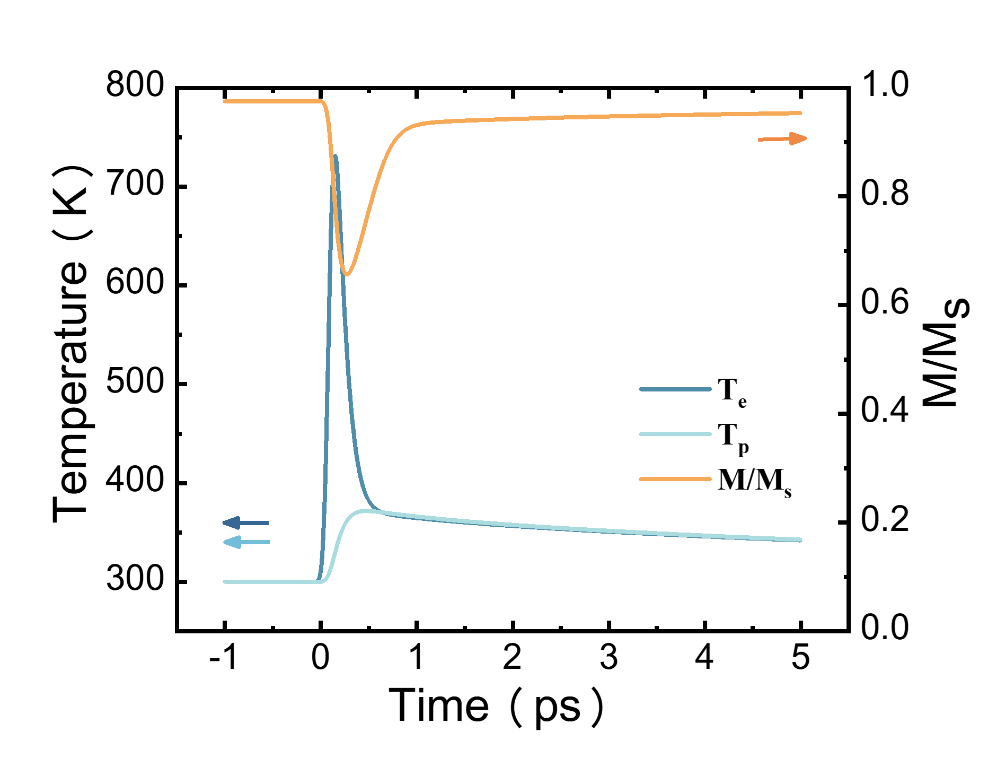


**Fig. S2 Simulation of the FeGa film using the three-temperature model.** The dynamics of the electron temperature $T_{e}(t)$ (blue), lattice temperature $T_{p}(t)$ (light blue), and magnetization $M(t)$ (orange) are shown.

We model the transient process using the python toolbox *udkm1Dsim*, which allows creating one-dimensional structures. Since the three-temperature system coupling parameters of FeGa materials are missing and Ga contributes very little to the magnetic properties of the film, we adopt Fe single crystal material as the subsystem coupling parameter: $\gamma=0.1 J \mathrm{kg}^{-1}K^{-2}$, $C_{p}=532 J \mathrm{kg}^{-1}K^{-1}$, $R=25.3 \mathrm{ps}^{-1}$, $T_{c}=675 K$, $g_{ep}=4.05\times{10}^{18} J\left( {s m}^{3} K \right)^{-1}$, $n=3.679+i10.413$, and the optical penetration length is 8 nm. We set the thickness of FeGa film to 50 nm and the initial boundary conditions to room temperature (300 K). The laser pulse fluence is 5 mJ/cm^2^, and pulse width is 100 fs. The calculated results are shown in Fig. S2.

**S2.3. Magnetic-optical-faraday effects**

The magnetization of the ferromagnetic film breaks the time-reversal symmetry of the system. In the discussion of the magneto-optical effect, we temporarily neglect the contribution of internal stress to the dielectric tensor. Considering the symmetry, the magnetization of FeGa film is in-plane isotropic, even in a single wrinkle. Therefore, there is no net in-plane magnetic moment and the surface morphology can be ignored when analyzing the magneto-optical signal. Considering the out-of-plane magnetization (**M**= (0, 0, 1)), the system symmetry in the coordinate system belongs to the **4mm** magnetic point group.^[5]^ The magnetization-dependent dielectric tensor has the following form:

|  | $\varepsilon_{ij}=\varepsilon^{(0)}\delta_{ij}+\chi_{ijk}M_{k}.$ | (S11) |
| --- | --- | --- |

The structure of the third rank tensor $\chi_{ijk}$ is the magneto-optical susceptibility, which described phenomenologically by the symmetry of the film and its magnetization. Here, $\varepsilon_{11}=\varepsilon_{22}$ and $\varepsilon_{12}={-\varepsilon}_{21}$. During the demagnetization process, the magnetization is instantaneously weakened. In this case, the dielectric tensor becomes time-dependent

|  | ${\varepsilon_{ij}}^{'}\left( t \right)=\varepsilon^{\left( 0 \right)}\delta_{ij}+\chi_{ijk}M_{k}\left( t \right),$ | (S12) |
| --- | --- | --- |

here, $M_{k}\left( t \right)$ can be described by the three-temperature model (Eq. S10). Within our phenomenological treatment, we assume the response of the dielectric tensor to magnetization as instantaneous. The change in the dielectric tensor is given by

|  | ${\Delta\varepsilon}_{ij}\left( t \right)={\varepsilon_{ij}}^{'}\left( t \right)-\varepsilon_{ij}\left( t \right)=\chi_{ijk}{\Delta M}_{k}\left( t \right).$ | (S13) |
| --- | --- | --- |

This induces magnetic circular birefringence of the transmitted light. The refractive index of left- (right-) circularly polarized light can be expressed as:^[6]^

|  | $\left( \boldsymbol{n}\pm\frac{\boldsymbol{g}}{2n_{0}} \right)^{2}=n_{0}^{2}.$ | (S14) |
| --- | --- | --- |

The second term is the contribution of the magnetic moment of the film. The gyration vector $\boldsymbol{g}$ represents the magneto-optic coupling parameter, and $g_{k}=\alpha_{ijk}\varepsilon_{ij}$, where $\alpha_{ijk}$ is a third-order tensor that depends on the crystal symmetry and magnetization orientation. $n_{0}$ is the isotropic refractive index of FeGa film without net magnetization. $\boldsymbol{n}$ is the resulting refractive index for a circular polarization of light propagating through the magnetized material. It is a vector because its value depends on the polarization state. The ± sign in the equation denotes the handedness-dependent nature of the magneto-optic interaction. Specifically, the plus sign (+) corresponds to the right circular polarization (RCP) mode $n_{+}$, while the minus sign (–) corresponds to the left circular polarization (LHCP) mode $n_{-}$. The change in magnetization during demagnetization induces a corresponding change in the refractive index:

|  | $\Delta n=n_{+}-n_{-}=\frac{g}{n_{0}}\propto\Delta\varepsilon_{ij}.$ | (S15) |
| --- | --- | --- |

The difference in refractive indices for left and right circularly polarized light induces a rotation of the polarization plane of the transmitted light:

|  | $\theta_{prob-f}=\frac{\omega g}{2cn_{0}}t_{FeGa},$ | (S16) |
| --- | --- | --- |

where $\omega$ is the angular frequency of the detection light, $c$ is the speed of light in a vacuum, and $n_{0}$ is the refractive index in the absence of the field. $g$ denotes the projection of the gyration vector along the light propagation direction, and is associated with the off-diagonal element of the dielectric tensor.

**S2.4. Photoelastic effect**

In a wrinkled film, the contribution of internal stresses to the ultrafast dynamics must be considered. As we describe in Eq. S7, magnetostriction causes additional strain $\Delta\epsilon_{ij}=\sigma_{ij}(z)/E$ in the film. When the demagnetization effect occurs, orbital electrons are excited to become hot electrons, disrupting the steady-state interatomic potential. The instantaneous drop in magnetization reduces the strain $\Delta{\epsilon_{ij}}^{'}\left( t \right)$ induced by the magnetostrictive effect and changes with time. However, the lattice movement speed is slower than the electron excitation speed, so it is manifested as an increase in the potential energy of interatomic interactions. This change corresponds to a change in the electron temperature on the time scale (Eq. S8):

|  | $\Delta{\epsilon_{ij}}^{'}\left( t \right)\sim f\left( t \right)T_{e}\left( t \right),$ | (S17) |
| --- | --- | --- |

where $f\left( t \right)$ is the time-dependent transfer function. This process can be phenomenologically described as an equivalent displacement $u_{ij}\left( t \right)$:

|  | $u_{ij}\left( t \right)=\Delta\epsilon_{ij}-\Delta{\epsilon_{ij}}^{'}\left( t \right).$ | (S18) |
| --- | --- | --- |

Correspondingly, we can introduce the equivalent displacement tensor component into the dielectric tensor:^[6]^

|  | ${\Delta\varepsilon}_{ij}\left( t \right)=a_{1}u_{ll}\left( t \right)\delta_{ij}+a_{2}u_{ij}\left( t \right),$ | (S19) |
| --- | --- | --- |

where ${\Delta\varepsilon}_{ij}\left( t \right)$ is the time-dependent change in the dielectric tensor. $a_{1}$ and $a_{2}$ are elastic-optical constants. $u_{ll}$ is isotropic volumetric strain due to volume expansion, and $u_{ij}$ is anisotropic contribution from the strain tensor, including both normal and shear components. Similar to the Faraday effect, the change in dielectric constant induces additional birefringence, inducing a change in the optical polarization angle

|  | $\theta_{prob-e}\propto{\Delta\varepsilon}_{ij}\left( t \right).$ | (S20) |
| --- | --- | --- |

Note that the simplified model above assumes that there is no lattice dynamics in the process and that the optical response to changes in electron distribution is instantaneous.

**Supplementary Note 3: Material characterization**


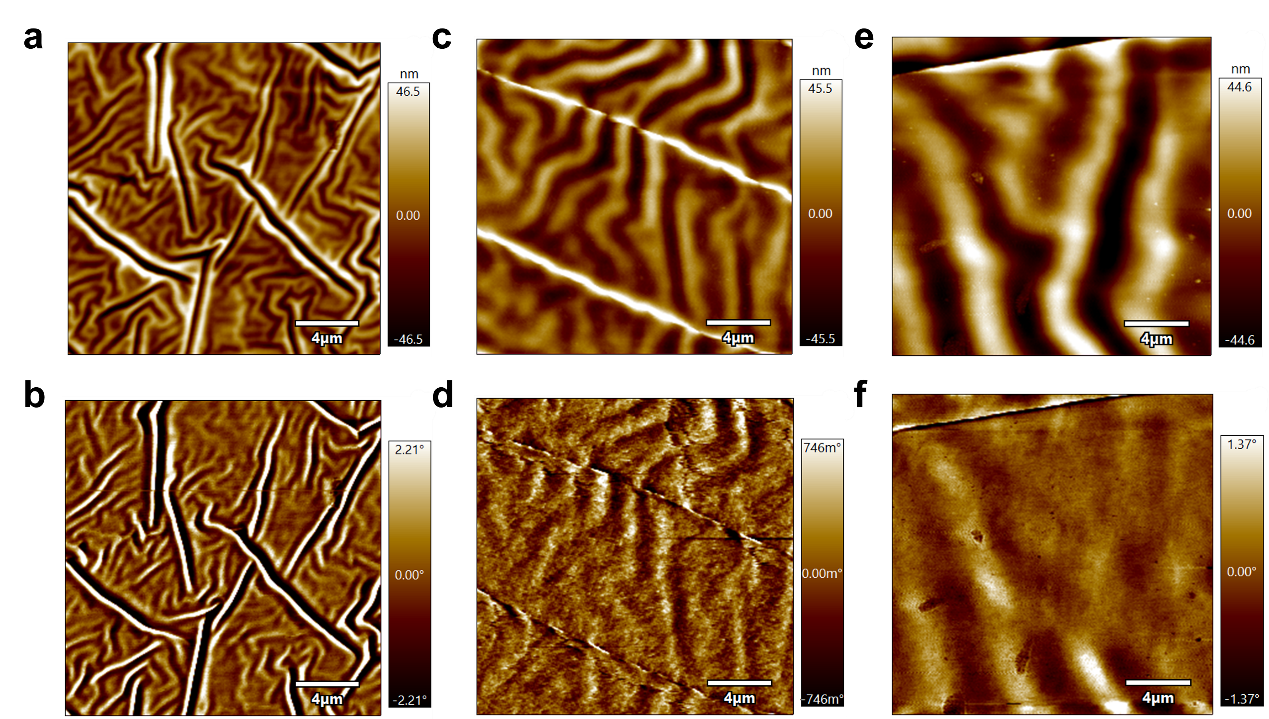


**Fig. S3** **Atomic force microscopy (AFM) and magnetic force microscopy (MFM) images.** The first row is the AFM images, and the second row is the MFM images. The three columns correspond to (**a, b**) FeGa(10 nm)/Pt, (**c, d**) FeGa(50 nm)/Pt and (**e, f**) FeGa(100 nm)/Pt respectively.

AFM and MFM measurements were performed on the samples with different FeGa thicknesses to investigate the relationship between film deformation and magnetization state. As shown in Fig.S3, the films exhibit distinct wrinkle morphologies, and the corresponding magnetic domain structures are spatially correlated with the wrinkle patterns. Tiny surface cracks can be clearly observed, which may arise from excessive internal stress within the substrate after sputtering, or from mechanical bending of the substrate during sample transfer.

Controlling the magnetization state of thin films via strain is a well-established approach,^[7, 8]^ particularly effective in giant magnetostrictive materials.^[9]^ The MFM results further reveal that opposite magnetization occurs in the “valley” regions between adjacent wrinkles, whereas the magnetic signal from the “peak” regions remains dominant. This behavior can be understood by considering the wrinkle formation process: wrinkles originate from substrate contraction during cooling after magnetron sputtering deposition. As the substrate shrinks, the peaks correspond to the film regions lifted by compressive stress, with the valleys remain attached to the substrate. Consequently, the vertical component of the magnetic moment at the wrinkle peaks provides the dominant contribution to the observed ultrafast signal.


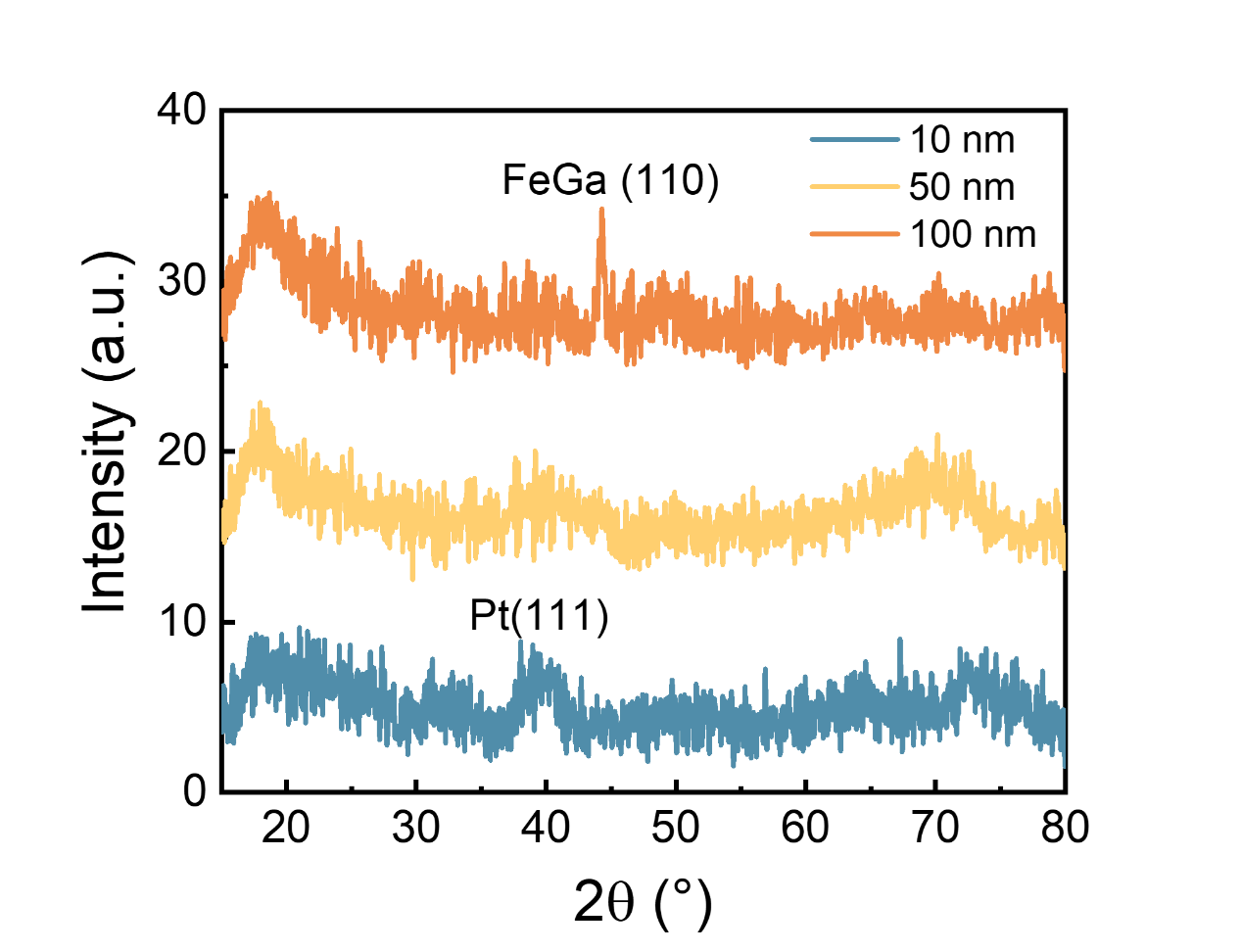


**Fig. S4** **X-ray diffraction (XRD) patterns of FeGa/Pt samples with different FeGa thicknesses.** Curves in different colors correspond to samples with varying FeGa layer thicknesses.

The XRD results indicate that the FeGa films are nearly amorphous, thereby ruling out significant magnetic anisotropy from crystallographic texture. This confirms that the observed anisotropy originates predominantly from the stress distribution rather than crystalline effects.

**Supplementary Note 4:** **Magnetization precession during relaxation**

**
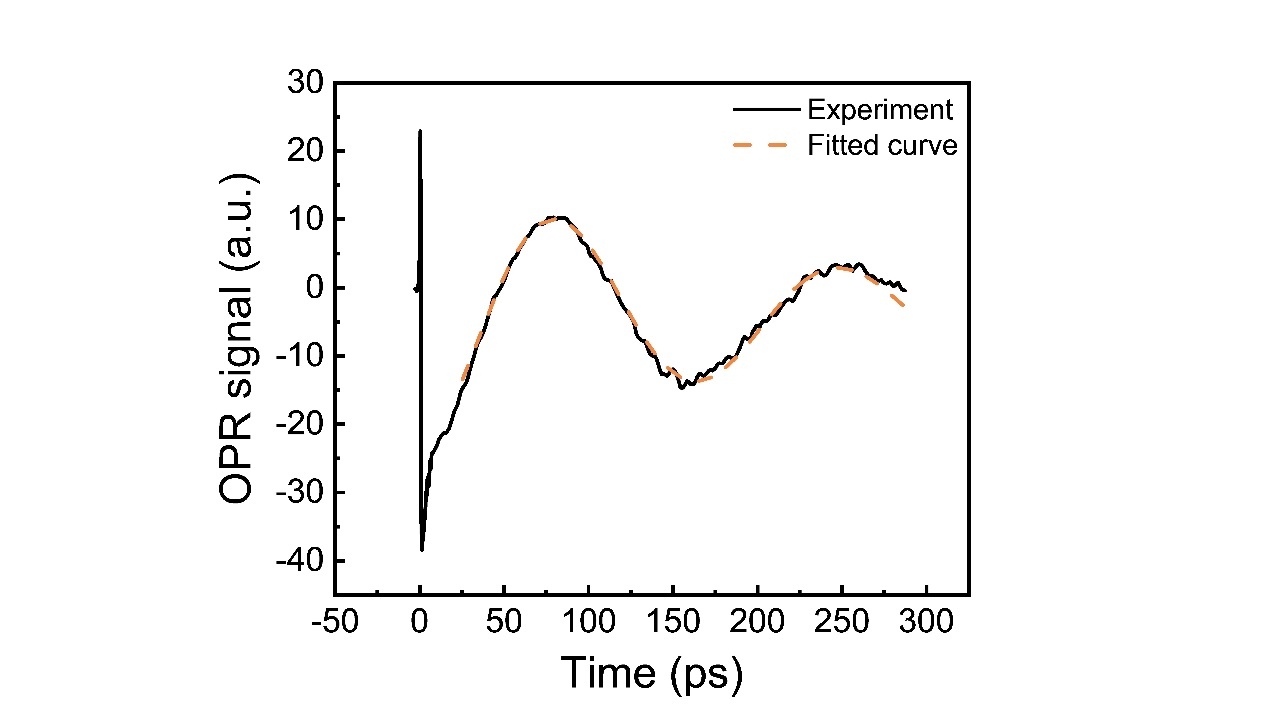
**

**Fig. S5 OPR raw signals when** $\boldsymbol{t}_{\boldsymbol{FeGa}}$**=50 nm with an external out-of-plane magnetic field (**$\boldsymbol{H}_{\boldsymbol{z}}$ **= 7000 Oe).** The black curve is the experimental data, and the orange dashed line is the fitting result.

The magnetic moment precession after demagnetization originates from the remagnetization process of the film. In magnetostrictive materials, magnetization precession is often accompanied by magnetoacoustic coupling. The coupled dynamics includes both coherent magnetization precession and natural decay caused by energy dissipation. This full process can be expressed as^[10]^:

|  | $\Delta M_{z}\left( t \right)=\Delta M_{z}^{max}\exp\left( -\frac{t}{\tau_{M}} \right)\sin\left( 2\pi ft-\psi_{0} \right)+Y_{0},$ | (S21) |
| --- | --- | --- |

where $\tau_{M}$ is the relaxation time, $f$ is the magnetic moment precession frequency, and $\psi_{0}$ is the initial phase. We fit the experimental results according to Eq. S21, as shown in Fig. S5. The excellent agreement between the data and the model indicates that the oscillatory signal with a 100 ps period is well-captured by the magnetic precession dynamics, thus unambiguously confirming its origin.

**Supplementary Note 5: The OPR signals when laser incident from PDMS substrate**


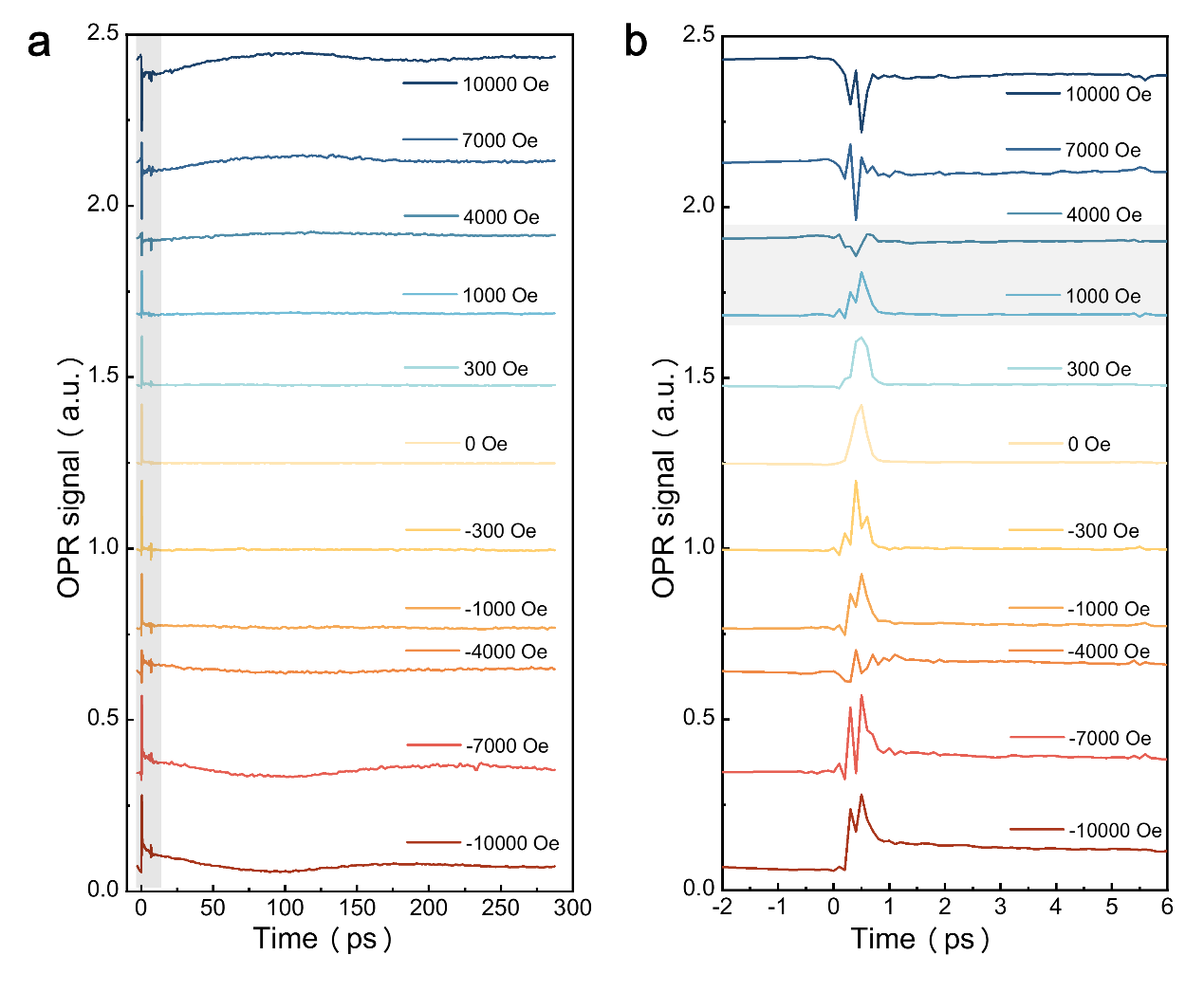


**Fig. S****6 OPR signals with laser incident from PDMS substrate. a,** OPR signals of the PDMS/FeGa(50 nm)/Pt(5 nm) structure under varying out-of-plane magnetic field. **b,** Enlarged view of the gray area in panel (**a**). The strain-induced signal exhibits a reversed sign within the light-grey region.

Fig. S6 shows the results when the laser is incident from the PDMS substrate. Upon reversing the sample, both the magnitude and the direction of the effective field, as indicated by the strain signal, are reversed. Since the substrate absorbs the laser pulse very weakly, the signal amplitude measured under this condition is stronger. Notably, multiple transient signals are observed. This may be due to the presence of several wrinkles within the probed area, coupled with the large surface roughness of the PDMS, which results in uneven stress and height distributions of the wrinkled film near the substrate. Consequently, multiple wrinkles may generate transient signals that overlap temporally.

Due to experimental limitations, we cannot determine the number or state of the wrinkles within the probed area. Nevertheless, despite the signal superposition, the evolution of the transient signal with the magnetic field remains clearly observable.

**Supplementary Note 6: COMSOL simulation: model setup and shear stress results**

The film dimensions were set to $1 \mu m\times1 \mu m\times t_{FeGa}$ ($t_{FeGa} = 20-100 \mathrm{nm}$). To simulate the bending behavior, one edge of the film's lower surface was fixed, and the opposite edge was displaced toward the center. All material parameters for the film were based on the actual properties of the FeGa film at room temperature: a Young's modulus $E$ of $144 GPa$ ^[11]^, a shear modulus $G$ of $58 \mathrm{GPa}$ ^[11]^, and a Poisson's ratio $v$ of $0.3$ ^[12]^. The mesh size was adapted to the film thickness, with finer meshes applied to thinner films. Upon completion of the simulation, the film's stress distribution was visualized as a color map, and the stress values along a reference line were extracted.


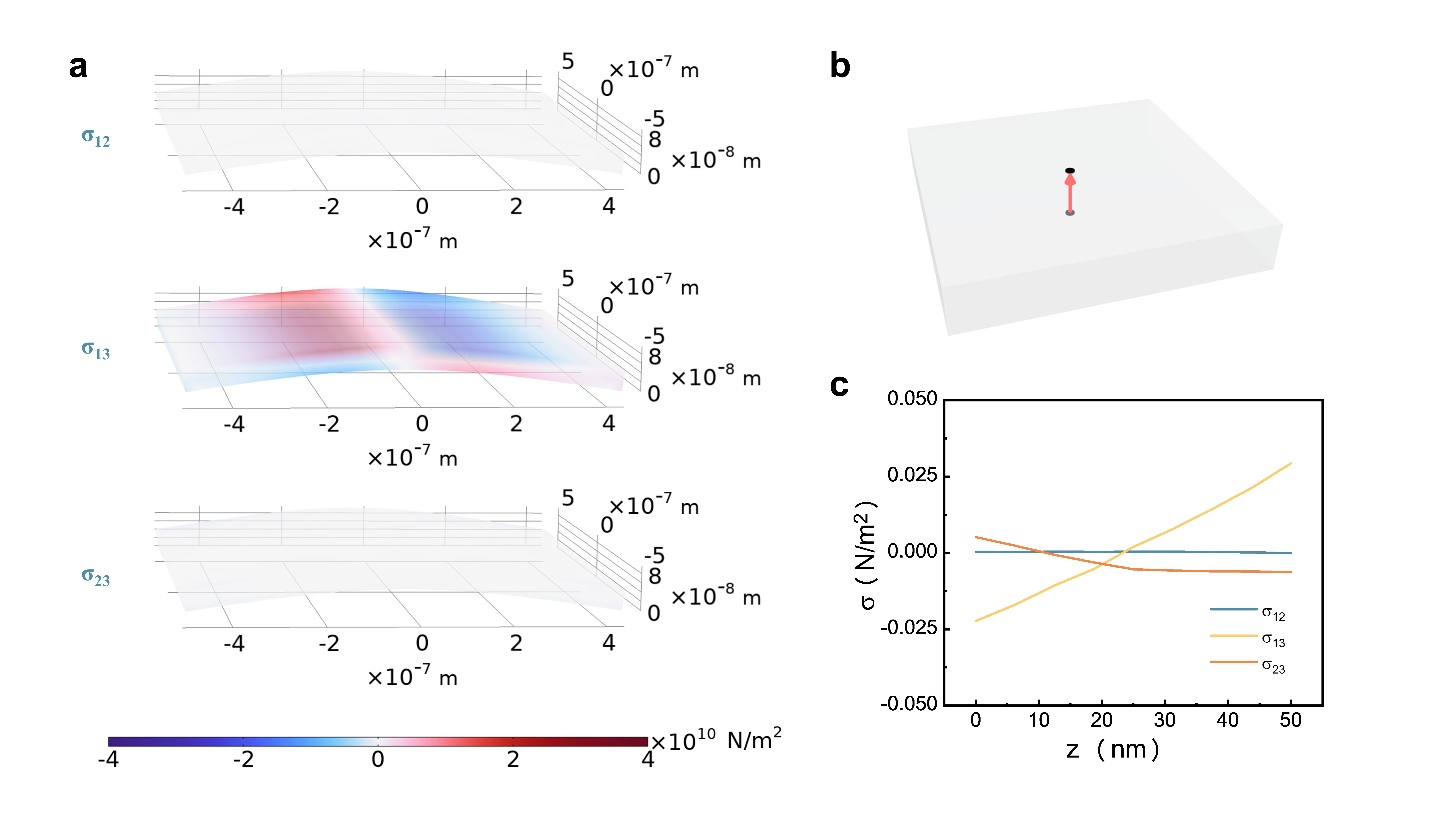


**Fig. S7 Shear stress in a bent film. a,** Shear stress components ( $\sigma_{12},\sigma_{13},\sigma_{23}$) in a 50 nm thick bent film with the unified color legend as the normal stress in Fig. 3. **b,** Selected reference line for stress output, located at the center of the film plane and pointing in the +z-axis direction. **c,** Shear stress distribution along the reference line.

Compared to the normal stress, the shear stress in the bent film is smaller by approximately ten orders of magnitude. Although $\sigma_{13}$ is relatively large, it exhibits an antisymmetric profile. Notably, the shear stress nearly vanishes at the central peak of the wrinkle. Therefore, the bias magnetic field within the film originates primarily from the normal stress.

**Supplementary Note 7: Substrate incident test results of** $\boldsymbol{t}_{\boldsymbol{FeGa}}$ **= 10 nm**


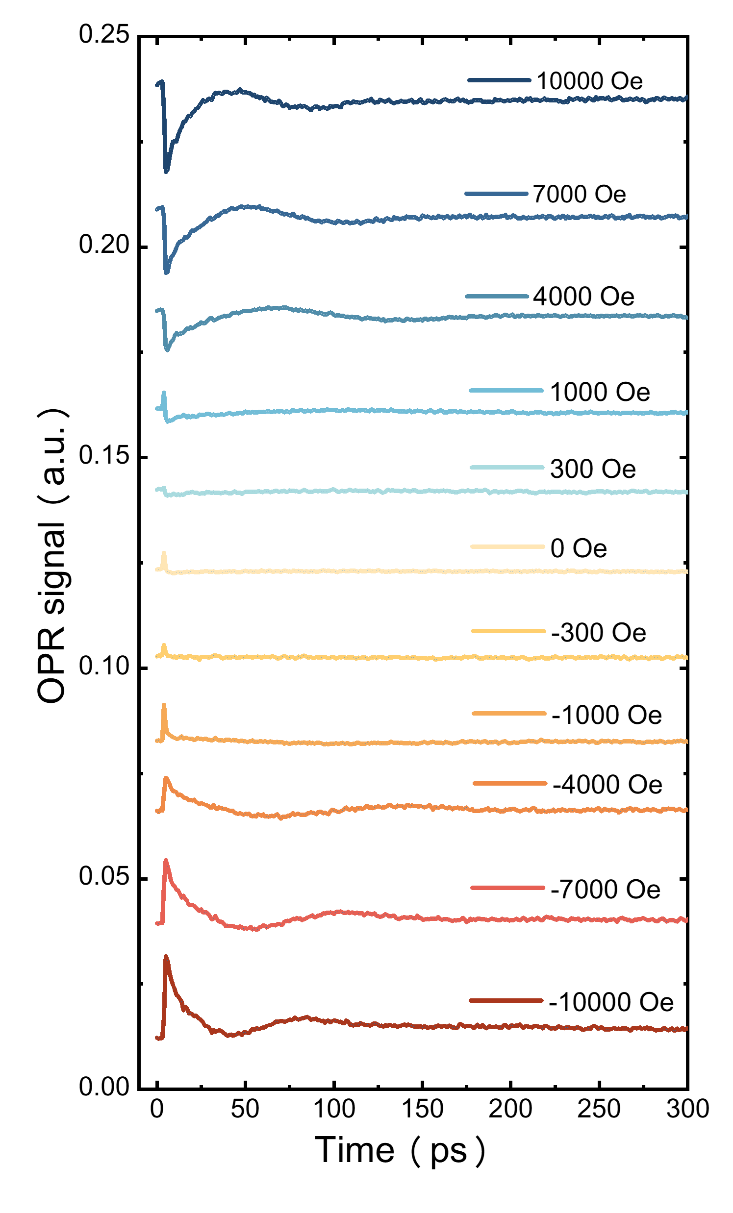


**Fig. S8** **OPR raw signals when** $\boldsymbol{t}_{\boldsymbol{FeGa}}$**=10 nm.** The laser is incident from the Pt surface.

For thinner FeGa films, the strain signal weakens and becomes detectable only under weak demagnetization. This observation aligns with COMSOL simulation results presented in the main text. Additionally, the substrate heating effect during film deposition is also reduced.

**Supplementary Note 8: Test results of PDMS substrate**


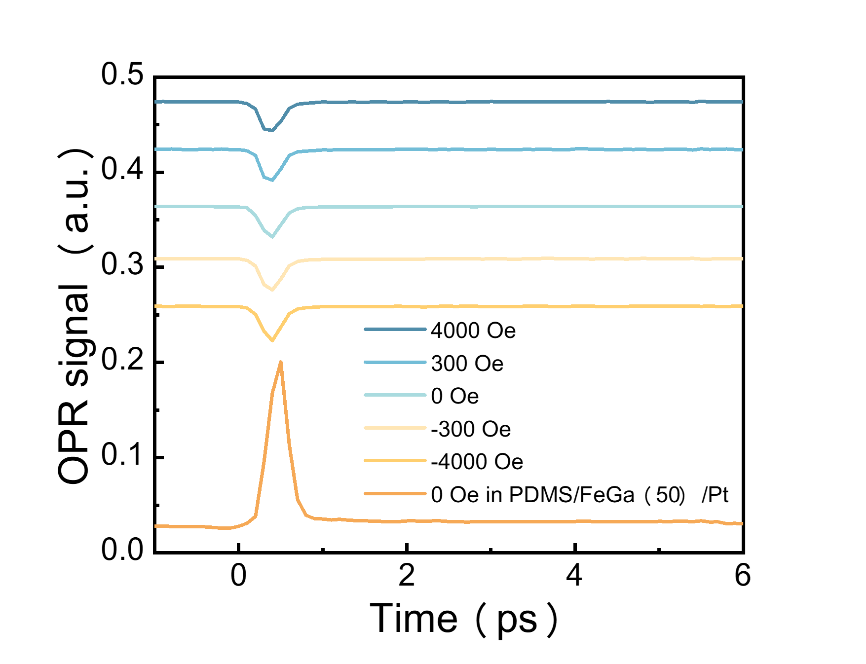


**Fig. S9 Comparison of OPR signals from the PDMS/FeGa(50 nm)/Pt structure and the PDMS substrate.** Shown are the signals of the PDMS substrate under varying out-of-plane magnetic fields and the sample strain signal in the absence of an external magnetic field.

To exclude the influence of the PDMS substrate, we measured its signal under different out-of-plane magnetic fields. The substrate signal remained unchanged with the applied magnetic field. In addition, its transient signal was significantly weaker than that of the PDMS/FeGa(50 nm)/Pt(5 nm) sample when the laser was incident from the substrate side. These results confirm that the transient signals observed in the experiment originates from the magnetization, i.e., the FeGa layer.

**Supplementary Note 9: Test results of MgO/FeGa(10 nm)/Pt**


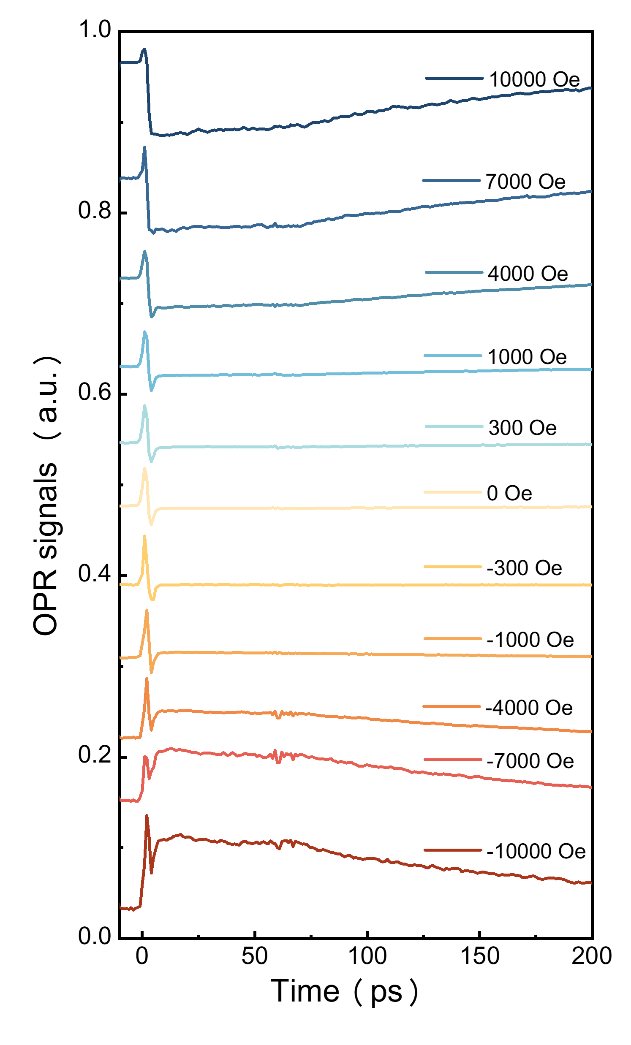


**Figure S10. OPR raw signals of the MgO/FeGa(10 nm)/Pt(5 nm) structure.** Light is incident from the film surface. The external magnetic field is applied out-of-plane.

**Supplementary Note 10: Transmission rate of PDMS/FeGa(50nm)/Pt**


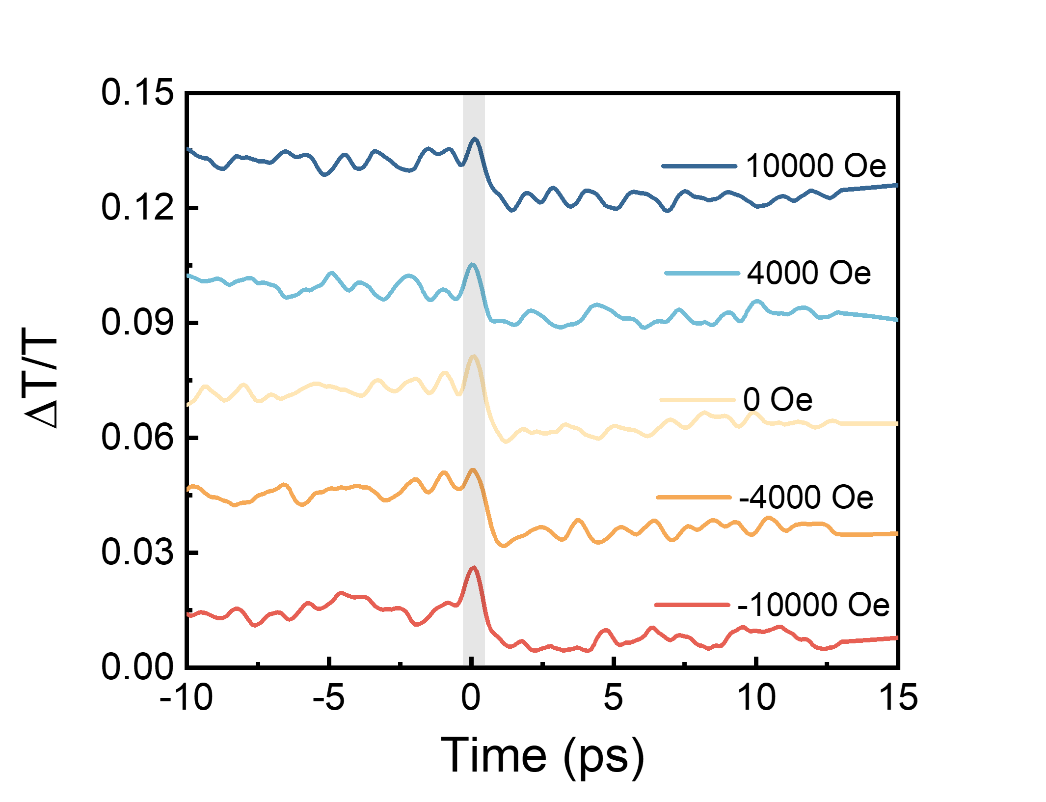


**Fig. S11 Transient transmission rate of PDMS/FeGa(50 nm)/Pt.** Light is incident from the film surface. The external magnetic field is applied out-of-plane.

**References**

[1] R. Wu, "Origin of large magnetostriction in FeGa alloys," *J. Appl. Phys.* 91 (2002): 7358.

[2] J. Niu, K. Yan, Y. Xu *et al.*, "Understanding the intrinsic mechanism of the giant magnetostriction in binary and alloyed FeGa solid solutions," *Phys. Rev. B* 109 (2024): 014417.

[3] J. M. Coey, *Magnetism and magnetic materials*. (Cambridge university press, 2010).

[4] B. Koopmans, G. Malinowski, F. Dalla Longa *et al.*, "Explaining the paradoxical diversity of ultrafast laser-induced demagnetization," *Nat. Mater.* 9 (2009): 259.

[5] R. Atkinson, N. Kubrakov, "Magneto-optical characterization of ferromagnetic ultrathin multilayers in terms of surface susceptibility tensors," *Phys. Rev. B* 66 (2002): 024414.

[6] L. D. Landau, J. S. Bell, M. Kearsley *et al.*, *Electrodynamics of continuous media*. (elsevier, 2013), vol. 8.

[7] C. Thiele, K. Dörr, O. Bilani, J. Rödel, L. Schultz, "Influence of strain on the magnetization and magnetoelectric effect in La_0.7_A_0.3_MnO_3_/PMN-PT (001)(A= Sr, Ca)," *Physical Review B—Condensed Matter and Materials Physics* 75 (2007): 054408.

[8] Z. Zhou, X. Cheng, M. Hu *et al.*, "Manipulation of the altermagnetic order in CrSb via crystal symmetry," *Nature* 638 (2025): 645.

[9] S. Bandyopadhyay, J. Atulasimha, A. Barman, "Magnetic straintronics: Manipulating the magnetization of magnetostrictive nanomagnets with strain for energy-efficient applications," *Appl. Phys. Rev.* 8 (2021): 4.

[10] V. N. Kats, T. L. Linnik, A. S. Salasyuk *et al.*, "Ultrafast changes of magnetic anisotropy driven by laser-generated coherent and noncoherent phonons in metallic films," *Phys. Rev. B* 93 (2016): 214422.

[11] S. Jen, W. Cheng, F. Chiang, "Structural, magneto-mechanical, and damping properties of slowly-cooled polycrystalline Fe_81_Ga_19_ alloy," *J. Alloys Compd.* 651 (2015): 544.

[12] G. Dai, Q. Zhan, Y. Liu *et al.*, "Mechanically tunable magnetic properties of Fe_81_Ga_19_ films grown on flexible substrates," *Appl. Phys. Lett.* 100 (2012): 122407.
